# Brain Responses to Faces and Facial Expressions in 5-Month-Olds: An fNIRS Study

**DOI:** 10.3389/fpsyg.2019.01240

**Published:** 2019-05-29

**Authors:** Renata Di Lorenzo, Anna Blasi, Caroline Junge, Carlijn van den Boomen, Rianne van Rooijen, Chantal Kemner

**Affiliations:** ^1^Experimental Psychology, Helmholtz Institute, Utrecht University, Utrecht, Netherlands; ^2^Developmental Psychology, Utrecht University, Utrecht, Netherlands; ^3^Department of Medical Physics and Biomedical Engineering, University College London, London, United Kingdom; ^4^Center Rudolf Magnus, University Medical Centre, Utrecht, Netherlands

**Keywords:** functional near-infrared spectroscopy, infancy, emotion processing, face processing, right hemisphere

## Abstract

Processing faces and understanding facial expressions are crucial skills for social communication. In adults, basic face processing and facial emotion processing rely on specific interacting brain networks. In infancy, however, little is known about when and how these networks develop. The current study uses functional near-infrared spectroscopy (fNIRS) to measure differences in 5-month-olds’ brain activity in response to fearful and happy facial expressions. Our results show that the right occipital region responds to faces, indicating that the face processing network is activated at 5 months. Yet sensitivity to facial emotions appears to be still immature at this age: explorative analyses suggest that if the facial emotion processing network was active this would be mainly visible in the temporal cortex. Together these results indicate that at 5 months, occipital areas already show sensitivity to face processing, while the facial emotion processing network seems not fully developed.

## Introduction

Learning to differentiate between facial expressions is vital for acquiring relevant social information. Previous research shows that infants’ ability to recognize human faces and to read emotions from faces undergoes rapid changes during the first year of life. Presumably, these changes in infant behavior are connected to changes in brain development (for reviews on how brain development links to face processing or facial emotion perception see Haist and Anzures, 2017, and Leppänen and Nelson, 2009, respectively). Neuroimaging studies in adults demonstrated that the brain circuits recruited during face and emotion processing are highly interacting (e.g., Vuilleumier and Pourtois, 2007). Yet little is known about how these brain networks emerge in infancy. To understand which parts of the face processing and facial emotion processing networks are activated in early development, the current study measured 5-month-olds’ brain activity while they viewed fearful and happy facial expressions, and houses as baseline stimuli.

In adults, evidence from functional brain imaging indicates that faces evoke specific activity in the so-called core face processing network. This network constitutes three cortical areas (Haxby et al., 2002): the fusiform gyrus (FG), the occipital face area (OFA) and the superior temporal sulcus (STS). Activity in these areas has been linked to different aspects of the processing of faces. In particular, the OFA is involved in the early perception of facial features, the FG in the processing of invariant aspects of faces, and the STS is associated with detecting changeable features of faces, such as lip movements. Although emotional faces might elicit activation from the “face processing” network as well, differentiation between emotions is typically observed in the “facial emotion processing” network, which comprises the STS, the amygdala, and the orbitofrontal cortex (OFC; Leppänen and Nelson, 2009). The amygdala rapidly responds to facial expressions and successively enhances processing in cortical face-sensitive areas (i.e., FG and STS; Leppänen and Nelson, 2009). Regarding the OFC, this region is presumably involved in emotion recognition and top-down modulation of perceptual processing (Leppänen and Nelson, 2009). In adults, the connection between face and facial emotion brain networks is considered to be bi-directional: the amygdala and OFC not only send information to cortical regions of the face processing network (FG and STS), but also receive information from these visual areas (Leppänen and Nelson, 2009). While the neural basis of face and facial emotion processing have been extensively studied in adults, little is known about which of these cortical areas are active in the infant brain already at 5 months of age.

In infancy, face categorization may possibly be innate: newborns preferentially orient towards face-like configurations rather than to non-face stimuli (Johnson et al., 1991). Studies using electroencephalography (EEG) also reveal differential processing of faces as young as 3 months of age (earliest age reported; e.g., Halit et al., 2004). Recently, functional near-infrared spectroscopy (fNIRS) has been used to measure changes in oxy- (HbO_2_) and deoxy-hemoglobin (HbR) concentrations to infer localized cortical activity in the infant brain, which is indexed by an increase in HbO_2_ concentration coupled with a decrease in HbR concentration (for a review on fNIRS see Lloyd-Fox et al., 2010). Since the haemodynamic responses are restricted to cortical brain regions where neuronal activation occurs, fNIRS, like fMRI, offers a higher spatial resolution than EEG. fNIRS has been successfully used to measure infant brain responses to face stimuli, with some studies observing a right hemispheric dominance already at 5 months of age (Nakato et al., 2009). Face-specific activity has been revealed in the occipital and temporal areas, suggesting activation of the face processing network (Blasi et al., 2007; Otsuka et al., 2007; Nakato et al., 2009, 2011a; Honda et al., 2010). However, this conclusion is based on only few studies, of which just two investigated this selectively in 4-to-5-month-olds (see research on occipital areas: Blasi et al., 2007; and occipito-temporal areas: Nakato et al., 2009). Moreover, none of these studies investigated face-specific responses using emotional faces. The conclusions thus benefit from replication and extension with such stimuli.

Facial emotion categorization also matures rapidly, albeit somewhat later than face processing. The field usually considers 7 months of age the tipping point at which infants respond differently to different emotional expressions. For instance, an EEG study showed that 7-month-olds discriminated between fearful and neutral or happy facial expressions (e.g., Leppänen et al., 2007; for an overview see van den Boomen et al., 2017). Yet two out of the four EEG studies on 4- to 5-month-olds suggest that this ability starts even earlier, by showing differential activity between happy and fearful faces (Rigato et al., 2009; Yrttiaho et al., 2014) while the other two reported no significant difference (Peltola et al., 2009; Hoehl and Striano, 2010). No study investigated facial emotion processing at this young age using fNIRS. In older infants, one fNIRS study suggested that at 7 months, the STS is involved in the processing of angry (right STS) and happy faces (left STS; Nakato et al., 2011b). Furthermore, pictures or videos of happy faces activate the OFC in 7- to 13-month-olds (Minagawa-Kawai et al., 2009; Fox et al., 2013; Ravicz et al., 2015). It remains uncertain whether temporal and frontal areas also differentially respond to different emotional expressions in infants as young as 5 months. As such, while some EEG studies suggest that part of the facial emotion processing network might be active at 5 months of age, no fNIRS study has explored which parts of this network might be involved.

The current study aimed to investigate which areas of the face and the facial emotion processing networks are active during early infancy. Therefore, we used fNIRS to study these processes in three locations: the occipital (close to the OFA), the temporal (close to the STS) and the frontal (close to the OFC) cortices. As EEG studies revealed that at 5 months of age face processing is possible, but facial emotion processing is still maturing, we studied infants at this age to capture the development of the networks at an early stage. We hypothesized that the face processing network is functioning, which would be reflected in activity at the occipital and temporal channels. This would replicate the results from previous fNIRS studies (Blasi et al., 2007; Otsuka et al., 2007; Nakato et al., 2009, 2011a; Honda et al., 2010). Analyses on activation of the facial emotion processing network were more explorative due to the absence of fNIRS studies and the conflicting results in EEG studies. Nevertheless, we expected that if (part of) this network was active, this would be reflected in activity in the temporal and frontal channels.

## Materials and Methods

### Participants

We tested 17 healthy 5-month-olds (9 girls; M_age_ = 163.4, range: 127–182 days). Two additional infants were excluded because they viewed fewer than three trials per condition (e.g., Lloyd-Fox et al., 2013). Both parents gave written informed consent prior to participation. The Medical Ethical Committee of the University Medical Center of Utrecht approved the study, which was conducted in accordance with the Declaration of Helsinki. Children received a book as a token for their participation.

### fNIRS Recording

**FIGURE 1:**
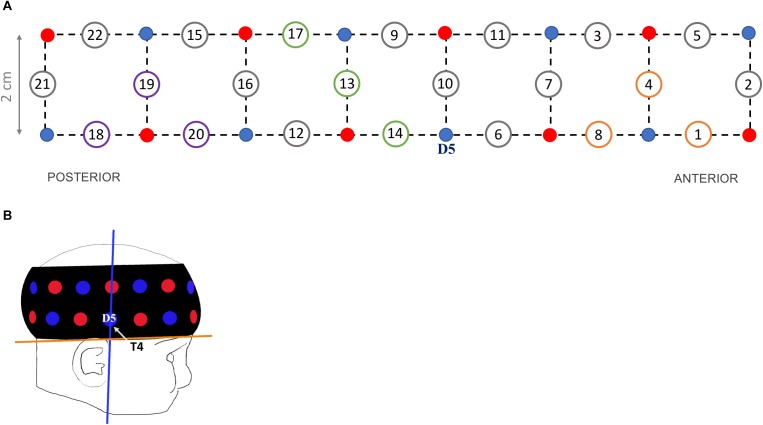
**(A)** Headgear design showing the position of sources (red dots), detectors (blue dots) and channels at a 2-cm source-detector separation (circled numbers). Channels of interests are 3 occipital (purple circles: 18, 19, 20), 3 temporal (green circles: 13, 14, 17) and 3 frontal (orange circles: 1, 4, 8). **(B)** Illustration of a 5-month-old wearing the probe band covering the right hemisphere, with the blue line corresponding to optimal vertical position of detector 5 (‘D5’, corresponding to electrode T4 in the 10–20 system) and the orange line to optimal horizontal position.

Haemodynamic responses were recorded at a 10 Hz sampling rate using the UCL topography half-system (NTS2; Everdell et al., 2005). Infants wore a fNIRS headgear consisting of eight dual-wavelength sources (780 nm, 850 nm) and eight detectors, which form 22 source-detector channels at a separation of 2 cm (Figure 1A). The probe array covered parts of the right occipital, temporal and frontal cortices (Lloyd-Fox et al., 2014).

To standardize the headgear position across participants, external scalp landmarks were used as reference (Blasi et al., 2014). The headgear was placed over the right hemisphere with detector 5 centered above the pre-auricular point (T4 according to the 10–20 system; vertical axis in Figure 1B). The lower edge of the probe band was aligned to the line between the top of the ear lobe and the highest point of the eyebrow (horizontal axis in Figure 1B). The headgear position was checked before and after the experiment; photos were taken to review (shifts of) band placement. No child was excluded for incorrect NIRS sensor placement, defined as a shift of more than 1 cm on the horizontal or vertical axes from the reference point.

### Stimuli

**FIGURE 2:**
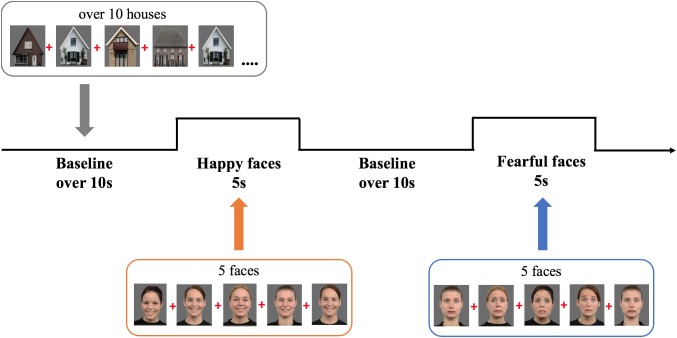
Experimental design showing the order and timing of stimulus presentation for the experimental (five pictures of female faces expressing either happiness or fear) and baseline trials (at least 10 s of pictures of houses). Face stimuli are obtained from the Radboud Faces Database (Langner et al., 2010). The depicted individuals provided written informed consent for the publication of their identifiable images.

We selected six female models expressing fear or happiness from the Radboud Faces Database (see Figure 2; identities: 12, 22, 26, 27, 37, 61; Langner et al., 2010). Baseline stimuli were 12 pictures of houses selected from the internet. The colored images were depicted on a gray background (RGB: 108) and measured 20.5 cm width × 22.5 cm height (visual angle: 19.4° × 21.2°).

### Procedure

During the study infants sat on their parent’s lap, at 60 cm from a 23-inch computer monitor (refresh rate 60 Hz, 1920 × 1080 resolution), in a semi-dark room. Parents were instructed not to interact with their child during the experiment. A video camera placed on top of the screen recorded the child’s behavior. The task was programmed in Matlab using Psych-Toolbox 3 (Brainard and Vision, 1997). It comprised alternating 5-s experimental trials displaying sequences of five either happy or fearful expressions interleaved with baseline trials, of minimum 10 s, displaying sequences of at least 10 houses (*M* = 12.2 houses; *SD* = 0.69; range = 10–22; Figure 2 depicts the task design). Every stimulus was presented for 800 ms, followed by a 200 ms interstimulus interval presenting a fixation cross; the order of the stimuli was randomized within trials. While the task always started with a baseline trial, the order of experimental conditions (fearful and happy facial expressions) was counterbalanced across infants. The ending of a baseline trial was controlled by the experimenter, who continued to the next trial only when the infant was attending the screen. To make the task more interesting, non-social sounds were randomly played after every 1–2 pictures, which caused a short delay in the following stimulus onset, producing the jittering of stimulus presentation. Only during baseline trials, the experimenter would play additional sounds to redirect the child’s attention to the screen. The experiment lasted on average 5 min and ended after all 10 trials per condition were presented or when an infant became too restless.

### Data Processing

First, we coded infants’ looking behavior offline and scored their compliance with the study. We excluded trials when the infant looked at the screen for less than 60% of the trial duration. Infants completed an average of 7 trials per condition (*SD* = 2.40; fearful condition: range 3–10; happy condition: range 3–9 trials).

The fNIRS data were then processed with Homer2 (MGH-Martinos Center for Biomedical Imaging, Boston, United States). First, channels with very low or high intensity levels were discarded with the enPrunechannel function. Raw intensity data of the remaining channels were converted to optical density units. Spline interpolation (*p* = 0.99) and wavelet (iqr = 0.80) functions in Homer2 were used to correct for motion artifacts in the data. Motion artifacts were detected by the function hmrMotionArtifactByChannel (AMP = 0.40, SDThresh = 15.5, tMotion = 1.0, tMask = 1.0). We excluded trials with artifacts in the [-2 + 5 s] time window that survived the correction. The data was then band-pass filtered (0.030–0.80 Hz) and changes in concentration of HbO_2_ and HbR were calculated using the modified Beer–Lambert Law, with a pathlength factor of 5.1 (Duncan et al., 1995). Finally, we computed a block average for 2 s pre- and 15 s post-stimulus onset; the time window [-2 – 0 s] was used for baseline correction.

We selected three channels to encompass each region of interest, based on a NIRS-MRI co-registration scalp anatomical map of 4- to 6-month-olds (Lloyd-Fox et al., 2014) and on the 10–20 system coordinates typically used in EEG. For the occipital region we selected channels 18, 19, and 20 located approximately between T6 and O2 (channels nearest to the OFA region); for the temporal region channels 13, 14, and 17 (surrounding the STS region); and for the frontal region channels 1, 4, and 8 (corresponding approximately to the OFC region). For each infant, the haemodynamic responses of every ROI were calculated by averaging the responses across the channels within each region, separately for each condition.

### Statistical Analyses

Maximum haemodynamic changes (peak amplitudes) were calculated for both HbO_2_ and HbR. Note that typically, in infant research, HbO_2_ is the preferred measure of activation, as it has higher SNR and is more consistent compared to HbR concentration change (for a more in depth discussion see Lloyd-Fox et al., 2010). Therefore, we will focus the discussion on the HbO_2_ results. To investigate differences in the timing of the response across experimental conditions (Nakato et al., 2011b) we selected two relatively narrow time windows for statistical analyses: 3–8 and 8–13 s post-stimulus onset. These periods of time were chosen to include the range of maximum concentration changes observed across infants for HbO_2_ and HbR, based on visual inspection of the grand-averaged hemodynamic responses and in accordance with previous work published with a similar task (e.g., Lloyd-Fox et al., 2013, 2017). Previous research that tested a similar age group with face stimuli showed that the haemodynamic response is expected to peak after the end of the experimental trial (e.g., Lloyd-Fox et al., 2017).

Statistical tests were performed using IBM SPSS Statistics 25.0 (IBM Corporation, Armonk, NY, United States). We carried out separate 3-way omnibus repeated measures ANOVAs to investigate changes in HbO_2_ or HbR in response to different facial expressions of emotion (fearful and happy) across the regions of interest (Temporal, Frontal, and Occipital) and across the two Time Windows. Overall, this analysis captures both effects of face processing and emotion processing. In particular, face processing is indexed by main effects of ROI or Time Window, which correspond to differences in the activity evoked by face stimuli between regions or in timing, respectively. At the same time, emotion discrimination is reflected by any main effect or interaction with emotional expression resulting from the ANOVA. Any significant effects were followed up with paired and one-sample *t*-tests. Since no prior fNIRS study tested emotion discrimination in 5-month-olds, a set of exploratory analyses were run to look at effects of happy vs. fearful expressions at each of the three ROI, for both HbO_2_ and HbR values, even in the case of an absence of significant interaction of emotion and ROI.

We report additional one-sample and paired *t*-tests (channel by channel, for HbO_2_ and HbR) in the Supplementary Table S1, as these analyses are more commonly reported in infant fNIRS studies. Overall, uncorrected analyses revealed significant effects of fearful and happy faces on HbO_2_ in the occipital and temporal, but not frontal regions. However, these results did not survive FDR correction for 22 comparisons.

## Results

**FIGURE 3:**
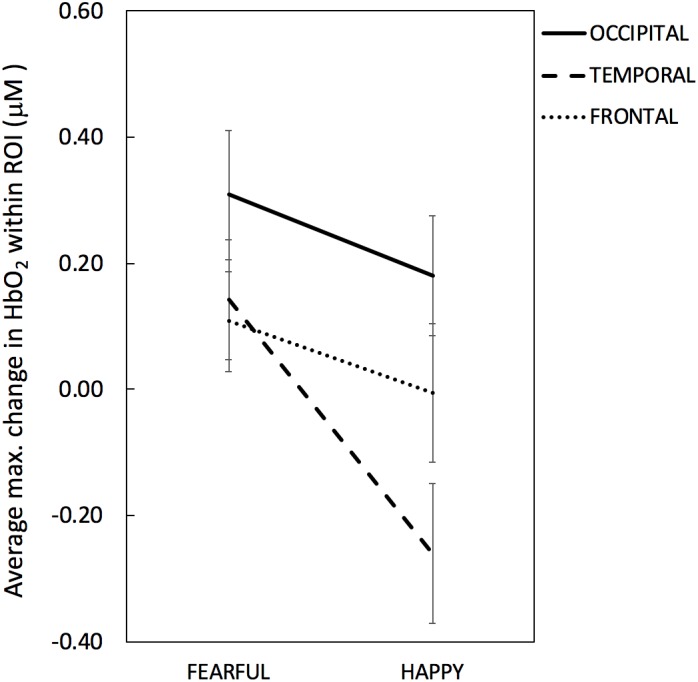
Maximum change (or peak amplitude) in HbO_2_ averaged across channels within each ROI per emotional condition (Fearful, Happy). The solid line denotes occipital ROI, the dashed line denotes temporal ROI and the dotted line denotes frontal ROI. Error bars represent standard error of the mean.

Figure 3, 4 depict the haemodynamic responses to happy and fearful faces. The ANOVA on HbO_2_ activity yielded a significant main effect of ROI [*F*(2,32) = 3.67, *p* = 0.037, η^2^ = 0.19]. Follow-up paired sample *t*-tests revealed that HbO_2_ was significantly higher at Occipital (*M* = 0.24, *SD* = 0.36) than Temporal regions (*M* = -0.059, *SD* = 0.24; *t*(16) = 2.9, *p* = 0.010, *d* = 0.70), while no difference was found between Frontal (*M* = 0.051, *SD* = 0.32) vs. Occipital [*t*(16) = -1.52, *p* = 0.15, *d* = 0.39], or Frontal vs. Temporal regions [*t*(16) = 1.03, *p* = 0.32, *d* = 0.25]. Yet, the one sample *t*-tests assessing whether the face response deviated significantly from zero indicated that only for the Occipital region face stimuli elicited a significant increase in HbO_2_ [*t*(16) = 2.8, *p* = 0.012, *d* = 0.68]; no other difference was found significant [Temporal: *t*(16) = -1.01, *p* = 0.33, *d* = 0.24; Frontal: *t*(16) = 0.66, *p* = 0.52, *d* = 0.16].

**FIGURE 4:**
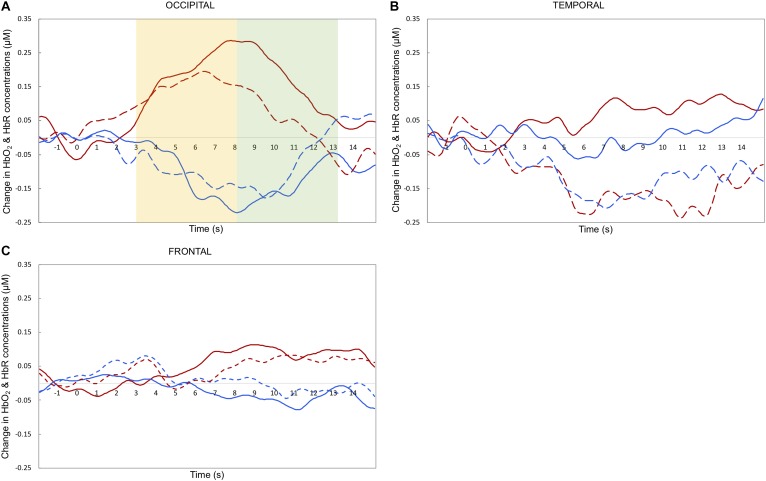
Grand averaged haemodynamic responses (in μM) to happy (dashed line) and fearful (solid line) faces, recorded from occipital **(A)**, temporal **(B)**, and frontal **(C)** regions. HbO_2_ is in red and HbR is in blue. The yellow and green areas in the upper left panel highlight the early and late time windows, respectively; *t* = 0 indicates stimulus onset.

There was a marginal effect of Emotion [*F*(1,16) = 4.06, *p* = 0.061, η^2^ = 0.20] reflecting a trend towards higher HbO_2_ values for Fearful (*M* = 0.19, *SD* = 0.26) than Happy expressions (*M* = -0.028, *SD* = 0.28), and a marginal interaction between Emotion and ROI [*F*(2,32) = 3.17, *p* = 0.055, η^2^ = 0.17]. No other significant effects were found. The ANOVA on HbR values yielded no significant effects (max *F* = 3.10; min *p* = 0.097).

Based on the explorative nature of the investigation of facial emotion processing at this age and the marginal effect of Emotion and marginal interaction between Emotion and ROI, we further looked into emotion responses in each region to help guide future studies. The paired *t*-test performed on HbO_2_ values revealed that only for the Temporal region HbO_2_ was significantly higher for Fearful (*M* = 0.14, *SD* = 0.39) than Happy faces (*M* = -0.26; *SD* = 0.45), *t*(16) = 2.38, *p* = 0.030, *d* = 0.59; no difference between facial expressions was found for the Occipital (Fearful: *M* = 0.31, *SD* = 0.43; Happy: *M* = 0.18, *SD* = 0.39; *t*(16) = 1.34, *p* = 0.20, *d* = 0.32), nor for the Frontal areas (Fearful: *M* = 0.11, *SD* = 0.33; Happy: *M* = -0.005, *SD* = 0.45; *t*(16) = 0.99, *p* = 0.34, *d* = 0.25). Follow-up one-sample *t*-tests performed for the Temporal HbO_2_ values indicated that the effect of Emotion was due to HbO_2_ non-significantly increasing for Fearful faces [*t*(16) = 1.51, *p* = 0.15, *d* = 0.38] while significantly decreasing for Happy faces [*t*(16) = -2.36, *p* = 0.031, *d* = -0.59]. The exploratory paired *t*-tests examining emotion differences computed on HbR values yielded no significant difference between facial expressions in any ROI (max *t* = 1.28, min *p* = 0.22).

## Discussion and Conclusion

This research aimed to investigate which cortical areas are sensitive to basic face processing and explore which ones are involved in facial emotion processing in 5-month-old infants. Overall, our results indicate that the right occipital area responds to faces regardless of emotional expression. A marginal emotion by region interaction seemed to relate to differences between facial emotions in the temporal region. Surprisingly, explorative analyses revealed that happy facial expressions elicit a significant decrease in HbO_2_ at temporal sites, while HbO_2_ concentrations followed the opposite direction in the fearful condition (albeit not significantly above zero). Generally, these results suggest that the face processing network is active in 5-month-olds, while activity in the facial emotion processing network appears not yet mature.

Our findings on the involvement of occipital areas in general face processing align with previous infant fNIRS research: a significant increase in HbO_2_ in response to faces. For instance, one study with 4-month-olds reports that compared to visual noise, neutral faces reveal more widespread HbO_2_ increases in occipital channels (Blasi et al., 2007). Similarly, another study with 5-month-olds observes that neutral faces elicit HbO_2_ increases in channels placed between T6 and O2 (Nakato et al., 2009), although note that the authors attribute this activation to the nearby region of STS. The current study adds to these results that, at 5 months of age, the occipital cortex is also sensitive to faces with different emotional expressions, while the temporal and frontal regions do not show such sensitivity (note that the activity recorded at the occipital region did not differ from that at the frontal region as the latter showed a small but non-significant HbO_2_ increase). This occipital region is also close to a cortical area involved in face processing in the adult brain: the OFA. This region appears to be involved in the early perception of facial features; previous adult fMRI studies report an increased activity for faces versus other objects (Haxby et al., 2002). Therefore, we interpret this occipital activation as reflecting OFA response to facial features common to both facial expressions. In the temporal and frontal regions we did not observe such increases of HbO_2_ irrespective of facial expressions. Thus, our study provides further support that infants at this age can recruit occipital regions for basic face processing.

Next, we turn to our exploration of whether different facial expressions elicit different patterns of cortical activity in 5-month-olds. Our results suggest that only temporal sites show a possible sensitivity to different facial emotions, while the frontal regions did not. Based on the marginal results, no strong conclusion can be drawn. Possibly, the infant brain is not yet sensitive to fearful and happy facial emotions at 5 months of age. This suggestion is consistent with findings of some EEG studies (Peltola et al., 2009; Hoehl and Striano, 2010) and with the Leppänen and Nelson (2009) model on maturation of facial emotion processing, confirming that the network is still immature at 5 months of age. Possibly the infant brain requires additional exposure to different types of emotions before the relevant brain areas become engaged with processing emotions. Another possibility is that the facial emotion processing network already shows some sensitivity to emotional expressions. This option in is line with other EEG studies (Rigato et al., 2009; Yrttiaho et al., 2014). The current results reveal that if this is the case, the temporal region is the first cortical area of the network to become active. Although the temporal region is where one would expect to differentiate between emotions, our results do not show the predicted pattern. Instead, we observe a strong HbO_2_ decrease for happy expressions, coupled with a non-significant HbO_2_ increase for fearful expressions.

What can a decrease in HbO_2_ reflect? The general consensus in the fNIRS community is that an HbO_2_ decrease cannot be interpreted as cortical activation; only an increase of HbO_2_ and/or a decrease in HbR are considered markers of brain activity, with increases of HbO_2_ being the most favored measure of activation in infant research (for discussion see Lloyd-Fox et al., 2010). Nevertheless, there have been a number of infant studies that occasionally report significant decreases in HbO_2_. Ravicz et al. (2015) reported such a decrease evoked by happy faces in frontal channels in 7-month-olds as well. They suggest there may be multiple potential causes for an HbO_2_ decrease: (i) it could be related to an unsteady and immature neurovascular coupling in infancy (Kozberg et al., 2013); (ii) it could also indicate neural deactivation, with the blood supply from that region being diverted to nearby areas that require oxygen (Wilcox et al., 2009); (iii) or it might well be a deactivation compared to baseline. However, these suggestions cannot fully explain our pattern of results: for instance, it is unlikely that neurovascular coupling is immature for happy but not for fearful faces, or that activation in the occipital region leads to a deflux of oxygen in the temporal region for happy faces only. Clearly, it remains puzzling why infant studies sometimes encounter decreases in HbO_2_ for one condition but not for another. More research is required to elucidate when and why this decrease in HbO_2_ appears before we can fully interpret the haemodynamic pattern observed in the temporal region.

It is also possible that with a larger sample size our weak findings of differential activation for the temporal region become clearer towards either direction of significance. It is important to note that even though we have a small sample size, our study matches the sample sizes typically employed in infant fNIRS studies (for an overview see Lloyd-Fox et al., 2010). With small sample sizes even small individual differences could mask group effects. Also, it could be that this individual variation is meaningful, and can be traced back to differences in brain maturation, every-day experience, or personality (e.g., Ravicz et al., 2015). More research is needed to understand the scope of individual variation in fNIRS infant studies. One further limitation of this study is that although we extensively researched the involvement of different regions in the right hemisphere, we cannot draw any conclusions on the involvement of the left hemisphere. The reason why we focus on the right hemisphere is because several studies suggest that this hemisphere is dominant for face processing even in infancy (e.g., Otsuka et al., 2007; Nakato et al., 2009). However, one infant study with 7-month-olds reveals that the left STS responds more to happy than to angry facial expressions (Nakato et al., 2011b). More research is required to fully understand the contribution of the left temporal region for emotion discrimination.

In summary, the present study provides further evidence that at 5 months of age infants recruit occipital areas while viewing faces, but also highlights that their cortical emotion-encoding mechanisms are still immature. Further work is needed to disentangle when and how across development the infant brain becomes able to discriminate between facial expressions.

## Ethics Statement

This study was carried out in accordance with the recommendations of the Medical Ethical Committee of the University Medical Center of Utrecht, with written informed consent from each infant’s parent. Both parents gave written informed consent in accordance with the Declaration of Helsinki prior to participation. The protocol was approved by the Medical Ethical Committee of the University Medical Center of Utrecht.

## Author Contributions

RDL and CB contributed in the conception of the study. RDL and RR created the task script and collected the data. Data processing was performed by RDL in collaboration with AB. RDL computed data analyses and wrote the first draft of the manuscript. All authors contributed in the revision of the manuscript and approved the submitted version.

## Conflict of Interest Statement

The authors declare that the research was conducted in the absence of any commercial or financial relationships that could be construed as a potential conflict of interest.
