# Supplementary material for: Brain Responses to Faces and Facial Expressions in 5-Month-Olds: An fNIRS Study
Source: Front Psychol. 2019 May 29;10:1240. doi: 10.3389/fpsyg.2019.01240 (PMC6548858; doi:10.3389/fpsyg.2019.01240)
Supplement: Supplementary file 1 [file Table_1.DOCX]

Supplementary Material

Brain responses to faces and facial expressions in 5-month-olds: an fNIRS study

Renata Di Lorenzo, Anna Blasi, Caroline Junge, Carlijn van den Boomen, Rianne van Rooijen, Chantal Kemner

*** Correspondence:**Corresponding Author: Renata Di Lorenzo
email: [renata.dlorenzo@gmail.com](mailto:renata.dlorenzo@gmail.com)

# S1. Supplementary Analyses

We also analyzed our data channel-by-channel, by calculating one-sample (emotion condition versus zero) and paired t-tests (fearful versus happy). Table 1 summarizes the significant haemodynamic responses at p < .05 (HbO_2_ and HbR) to happy and fearful facial expressions for both time windows (i.e., early: 3-8s, late: 8-13s).

*One-sample t-tests:* When comparing the pattern of significant differences for each emotion type, we observe that for both the early and the late time windows there are more occipital and temporal channels showing a significant increase in HbO_2_ to fearful faces (early: three; late: three) as compared to happy faces (only one with an increase in the early time window for an occipital channel). Surprisingly, the happy condition elicited a significant HbO_2_ decrease from three temporal channels (9, 11, 17) during the early time window and from one channel (17) in the late time window. Further, there is one frontal channel (3) showing a significant decrease in HbR for fearful faces.

*Paired-Sample t-tests:* Results of the paired t-tests reveal that fearful facial expressions evoke greater responses compared to happy expressions in a temporal channel during the late time window: HbO_2_ channel 10: *t*(16) = 0.73, *p* = .01.

However, none of the results presented here survive the FDR correction.

|  | Channel | Measure | Condition | Region | p-value | tstat | df |
| --- | --- | --- | --- | --- | --- | --- | --- |
| TW 3-8 s |  |  |  |  |  |  |  |
|  | 14 | HbO_2_ | Fearful | Temporal | 0.035 | 2.30 | 16 |
|  | 18 | HbO_2_ | Fearful | Occipital | 0.030 | 2.39 | 16 |
|  | 19 | HbO_2_ | Fearful | Occipital | 0.027 | 2.43 | 16 |
|  | 9 | HbO_2_ | Happy | Temporal | 0.030 | -2.38 | 16 |
|  | 11 | HbO_2_ | Happy | Fronto-Temporal | 0.007 | -3.12 | 16 |
|  | 17 | HbO_2_ | Happy | Temporal | 0.045 | -2.17 | 16 |
|  | 18 | HbO_2_ | Happy | Occipital | 0.033 | 2.33 | 16 |
| TW 8-13 s |  |  |  |  |  |  |  |
|  | 3 | HbR | Fearful | Frontal | 0.014 | -2.76 | 16 |
|  | 10 | HbO_2_ | Fearful | Temporal | 0.016 | 2.70 | 16 |
|  | 12 | HbO_2_ | Fearful | Temporal | 0.007 | 3.13 | 15 |
|  | 18 | HbO_2_ | Fearful | Occipital | 0.024 | 2.50 | 16 |
|  | 17 | HbO_2_ | Happy | Temporal | 0.031 | -2.36 | 16 |

*Table 1.* Results from the one-sample t-test, channel-by-channel analysis. Significant increases or decreases of HbO_2_ and HbR concentrations are reported for both conditions (*p* < .05). TW indicates the time windows selected in this study.
